# Nutritional senolytics and senomorphics: Implications to immune cells metabolism and aging – from theory to practice

**DOI:** 10.3389/fnut.2022.958563

**Published:** 2022-09-08

**Authors:** Carla Luís, Ana T. Maduro, Paula Pereira, José João Mendes, Raquel Soares, Renata Ramalho

**Affiliations:** ^1^Department of Biomedicine, Faculdade de Medicina da Universidade do Porto, Porto, Portugal; ^2^i3S – Instituto de Investigação e Inovação em Saúde, Universidade do Porto, Porto, Portugal; ^3^Nutritional Immunology – Clinical and Experimental Lab (NICE Lab), Clinical Research Unit, Centro de Investigação Interdisciplinar Egas Moniz (CiiEM, U4585 FCT), Egas Moniz Higher Education School, Monte de Caparica, Portugal; ^4^Applied Nutrition Study Group (Grupo de Estudos em Nutrição Aplicada – G.E.N.A.-IUEM), Egas Moniz Higher Education School, Monte de Caparica, Portugal; ^5^Instituto Universitário Egas Moniz, Egas Moniz Higher Education School, Monte de Caparica, Portugal

**Keywords:** aging, immunometabolism, immunosenescence, inflammaging, nutritional senolytics, nutritional senomorphics, precision nutrition

## Abstract

Aging is a natural physiological process, but one that poses major challenges in an increasingly aging society prone to greater health risks such as diabetes, cardiovascular disease, cancer, frailty, increased susceptibility to infection, and reduced response to vaccine regimens. The loss of capacity for cell regeneration and the surrounding tissue microenvironment itself is conditioned by genetic, metabolic, and even environmental factors, such as nutrition. The senescence of the immune system (immunosenescence) represents a challenge, especially when associated with the presence of age-related chronic inflammation (inflammaging) and affecting the metabolic programming of immune cells (immunometabolism). These aspects are linked to poorer health outcomes and therefore present an opportunity for host-directed interventions aimed at both eliminating senescent cells and curbing the underlying inflammation. Senotherapeutics are a class of drugs and natural products that delay, prevent, or reverse the senescence process – senolytics; or inhibit senescence-associated secretory phenotype – senomorphics. Natural senotherapeutics from food sources – nutritional senotherapeutics – may constitute an interesting way to achieve better age-associated outcomes through personalized nutrition. In this sense, the authors present herein a framework of nutritional senotherapeutics as an intervention targeting immunosenescence and immunometabolism, identifying research gaps in this area, and gathering information on concluded and ongoing clinical trials on this subject. Also, we present future directions and ideation for future clinical possibilities in this field.

## Introduction

Aging is an inevitable physiological chain of events that ultimately affects all systems and organs (1). In an aging world that is more concerned with longevity (lifespan) rather than the quality of life (healthspan), the search for modulable factors that slow down this process has found enthusiasts in the research field (2, 3). In fact, cellular aging begins long before the chronological rate (4, 5). Aging has been defined as the time-dependent functional decline that affects living organisms and for which the following “hallmarks” have been proposed: altered intracellular communication, genomic instability, telomere attrition, epigenetic alterations, loss of proteostasis, deregulated nutrient-sensing, mitochondrial dysfunction, stem cell exhaustion, altered intracellular communication, and cellular senescence (6). Senescence, particularly at the level of immune cells, is responsible for the phenotypic alteration of the innate and adaptive immune system cells and a marked decline in their biological function (7–9). The recent COVID-19 pandemic has reinforced the importance of modulating the immune response as aging sets in, since the fragility, susceptibility, and severity of infection in older age groups were evident (10–12). Nutrients can play a major role in immune cell metabolism, and nutrition (and particularly immunonutrition) as part of host-directed therapies is gradually acquiring greater scientific value in this area (13). In this context, and despite the increasing research on aging, senescence, and metabolism, there is room for further research regarding natural senolytic and senomorphic agents, which can be framed in a category of nutritional senolytics and senomorphics (14–16). It is recognized that senolytics may delay, prevent, or even reverse the process of senescence and contribute to health span and even lifespan (17–19). In another line of action, senomorphics indirectly impair the senescence process, acting as senescence-associated secretory phenotype inhibitors, dissociated from the process of cell killing (18, 20). In the first category, we highlight Quercetin, Fisetin, Piperlongumine, and Curcumin; and in the latter one, Resveratrol, Kaempferol, Apigenin, and Epigallocatechin gallate (EGCG) (16, 21). While biochemical and metabolic processes underlying the process of immunosenescence are more or less uncovered, the clinical applicability and efficacy of this type of senotherapeutics need to be addressed. In this article, we concisely describe the latest knowledge on the impact of nutritional senolytics and senomorphics on immune cell metabolism, we gathered information on concluded and ongoing clinical trials on this subject, and we present future directions for future clinical possibilities in this field. We will not address the use of these agents in age-associated pathologies, as previous studies already addressed those issues (21, 22).

### Immunosenescence and immunometabolism

The process of senescence is hallmarked by the irreversible halting of the cell division process and the permanent growth arrest without apoptosis (1). This can be either a replicative or a cellular senescence process and accompanies living organisms as their lifespan increases (4). As discovered by Hayflick and Moorhead in 1961, cellular senescence regulates cell fate, as senescent cells have significant metabolic and functional differences from their healthy counterparts (23). Senescent cells (SCs) experience changes in homeostasis, with activation of signaling pathways such as nuclear factor kB (NF-kB) and increased secretion of pro-inflammatory cytokines, chemokines, proteases, growth factors, and lipids (24). These alterations reprogram cell secretory phenotype, conducing to a senescence-associated secretory phenotype (SASP) associated with inflammation in aging (inflammaging). In fact, NF-kB is one of the main intracellular pathways responsible for inducing the inflammatory response and the SASP induction (25–27). Accumulation of SCs is, therefore, a direct cause of the aging process, mainly by autocrine and paracrine alteration of physiological responses in the surrounding microenvironment through SASP (22).

**FIGURE 1:**
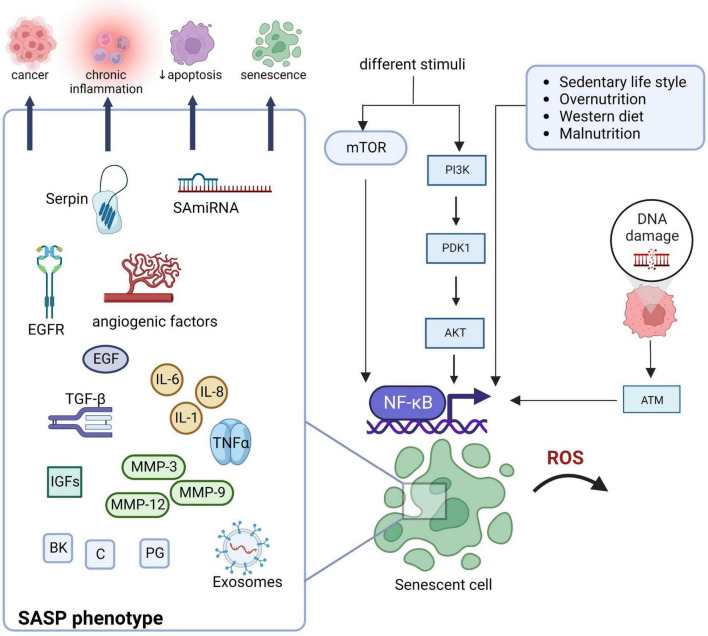
Schematic representation of main regulatory pathways activated in SCs (NF-kB, through mechanistic target of rapamycin -mTOR, and PI3k/Akt pathways) and production of proinflammatory and matrix-degrading mediator characteristics of SASP. SASP is related to increased chronic inflammation and other aging-associated conditions. Akt, protein kinase B; ATM, ataxia-telangiectasia mutated; BK, bradykinin; C, ceramides; EGF, epidermal growth factor; EGFR, epidermal growth factor receptor; IGFs, insulin-like growth factors; IL, interleukin; MMP, matrix metalloproteases; mTOR, mammalian target of rapamycin; NF-kB, nuclear factor-κB; PDK1, phosphoinositide-dependent kinase; Pi3K, phosphoinositide 3-kinases; PG, prostaglandin; SA-miRNA, senescence-associated microRNAs; TGF, transforming growth factor; TNF, tumor necrosis factor; ROS, reactive oxygen species. Created with BioRender.com.

In Figure 1, we present SASP components and the main regulatory pathway activated in SCs (the NF-kB, through mechanistic target of rapamycin -mTOR, and PI3k/Akt pathways), responsible for SASP and related to increased chronic inflammation and other aging-associated conditions (28, 29). In fact, as well-stated, NF-κB is crucial for inflammation and is subjected to complex redox regulation: either through the oxidation of cysteines in its DNA-binding region or through the oxidation of the inhibitor of NF-κB kinases (IκB) (30–33). Activation of NF-κB induces the gene transcription for inflammatory mediators such as IL-6 and IL-8. mTOR signaling is the main regulator through a positive feedback loop between NF-κB and interleukin (IL)-1α (30–33). It also promotes IL-1α translation that plays a pivotal role in SASP maintenance affecting the regulation of the NF-κB and C/EBP β DNA binding activities, inducing IL-6 and IL-8 transcription (34, 35). Control of SASP expression through the enhanced translation of MAPK-activated protein kinase 2 (MAPKAPK2 or MK2) has also been reported (34, 35). MK2 kinase is also activated upon stress through the activation of p38 mitogen-activated protein kinases (p38MAPK) and this promotes the activation of NF-κB (36, 37). Tumor necrosis factor (TNF)-α induces phosphorylation of AK kinase, resulting in signal transducers and activators of transcription 3 (STAT3) phosphorylation that translocate to the nucleus, contributing to persistent NF-κB activation and enhanced SASP expression (36) also promoted by the PI3k/Akt pathway. Persistent SASP expression induces a microenvironment of chronic proinflammation that promotes tissue dysfunction and cellular aging and other age-associated conditions (4, 30). Resistance to apoptosis, which plays a critical role in the removal of damaged and pre-neoplastic cells, is another hallmark of cellular senescence (36, 38).

As the aging process occurs, alterations in the immune response are expected – both in the innate and the adaptative systems (39–41). This state of altered immune response was named immunosenescence by Roy Walford in 1969 (42). The overall characteristics and evolution of this concept were elegantly reviewed elsewhere (7, 27, 43–45). Immunosenescence is affected by genetics, nutrition, previous encounters with microorganisms, biological and sex (although no differences between sexes were found regarding interventions targeting immunosenescence), and Human cytomegalovirus (HCMV) seropositivity (27). In Table 1, we synthesized the effect of aging on innate and adaptative immune systems. Aging is a malleable process, and the hallmarks of immunosenescence are influenced in a multifactorial way by both pharmacological therapies and nutrition; prompting the immune system as a target for interventions. The characteristics of immunosenescence are reduced ability to respond to new antigens, accumulation of memory T-cells, and low-grade inflammation; and these hallmarks are affected by individual exposure to pathogens during their lifetime [extensively reviewed in (27, 46)].

**TABLE 1:** Summary of effects of aging on innate and adaptative immune systems.

Senescent innate immune system	Senescent adaptative immune system
**Barriers – reduced response to antigens** ↓ skin cell replacement ↓ sweat production ↓ barrier function deterioration of epidermal immune response **Cellular components – reduced response to antigens, low-grade inflammation** *Overall* ↓ chemotaxis and phagocytosis, antigen presentation, cytotoxicity, activation of transduction signaling, and secretion of and response to cytokines *Dendritic cells* ↓ CD80/86 and MHC II ↓ INF-I/III production by pDCs ↓ TLR-mediated signaling by mDCs ↓ chemotaxis and endocytosis by mDcs ↓ antigen presentation by pDCs and mDCs *Macrophages* ↑ pro-inflammatory cytokines and PGE2 ↑ M1/M2 ratio ↓ chemotaxis and phagocytosis ↓ SuperOxide production (*oxidative burst*) ↓ TLR expression and function ↓ MHC II ↓ phagocytosis ↓ IL-2 production *Neutrophils* ↓ chemotaxis and phagocytosis ↓ SuperOxide production (*oxidative burst*) ↓ signaling transduction and apoptosis ↓ molecules recruitment into lipid rafts ↓ NETs Natural killer cells ↓ INF-γ	**Humoral response – reduced response to antigens** ↓ AID ↓ BCR repertoire ↓ IgM and IgD ↑ naïve B cells (CD19^+^ IgD^+^ CD27^–^ CD38^±^ CD24^±^) ↑ memory B cells (CD19^+^ CD27^+^ CD38^±^ CD24^±^) ↑ exhausted B cells ↑IgG and IgA **Cellular response – accumulation of memory T-cells** ↓ naïve T cells (CD45RA^+^ CCR7^+^ CD62L^+^ CD127^+^ CD132^+^ CD25^–^ CD44^–^ CD69^–^ CD45RO^–^ HLA-DR^–^) ↓ Th1 subsets ↓ signaling ↑ memory T cells ↑ CD28- T cells ↑ PD1 + Tim3 + T cells ↑ Th2 subsets

AID, activation-induced cytidine deaminase; BCR, B-cell receptor; DC, dendritic cell; IL, interleukin; INF, interferon; MHC, major histocompatibility complex; NETs, neutrophil extracellular traps; PGE, prostaglandin E; PD, programmed cell death protein; Th, T helper; Tim, T-cell immunoglobulin domain; TLR, toll-like receptor. Uparrow represent the increase.

Downarrow represent the descrease.

There is in fact considerable evidence that both cellular and humoral immunity are affected by aging (although, there is more published evidence demonstrating age-related changes to the adaptive immune system, particularly T-cells) determining a state of apparent immunosuppression and auto-reactivity (autoimmunity) (47–49). This is evidenced by the increased prevalence of infectious diseases in the elderly, both hospitalized and at home (11, 12, 50). Interestingly, cellular aging seems to be also accompanied by reinforcement of chronic low-grade inflammation by over-production of pro-inflammatory cytokines (IL-6, IL-1RA, TNF-α, IL-1, and C-reactive protein – CRP) by innate immunity – the previously reported inflammaging process (44, 51–54). The inflammatory environment that characterizes inflammaging is favored by the constant recognition of stimuli (mainly of metabolic, immunological, and hormonal nature) by innate immunity receptors (55).

If initially, the concept of inflammaging was revealed to be a direct consequence of immunosenescence, the bidirectional relationship between innate and adaptive cells makes it increasingly recognized that inflammaging is induced by immunosenescence and *vice versa* (56). This age-related inflammation is therefore associated with the increased risk of obesity (57), diabetes (58), cancer (59), and cardiovascular disease (60) (age-related inflammatory diseases), along with increased vulnerability to infection and decreased response to vaccination and frailty (52), as it was observed during COVID-19 pandemic (55). Lifelong exposure to pathogens led to several repetitive T-cell replications that ultimately transform into late-differentiated effector memory T-cells with features of replicative senescence and reduced proliferative activity (27). Inflammaging results from the activation of signaling networks critical to inflammation, such as those regulated by NF-kB, particularly when combined with other stimuli – like senescent cells, circulating mitochondrial DNA, obesity, gut microbiota, and diet (27), highlighting the importance of a specific, even-tailored (precision/personalized) dietary pattern.

Senescence has a profound effect on the metabolic regulation of immune cells, and the interest of the scientific community in this topic has grown (13). Immunometabolism refers to the cellular metabolism that shapes the outcome of cellular development and immune functions, impacting metabolic reprogramming on the immune response and immune cell’s fate (61–63). Cells’ immunometabolic profile directly influences activation, proliferative capacity, and quiescent state in tissues and in systemic circulation (13, 62). Not only does the functional specificity of each cell determine its biochemical needs, but each phenotype is influenced by factors such as pH, and nutrient and oxygen availability (64). Immunometabolism thus presents itself as essential for adequate immune function, since metabolic signaling regulates growth, proliferation, survival needs, and effector functions. Each immune cell subtype assumes different metabolic phenotypes to sustain energy balance supplies, biosynthesis demands, and longevity (36). For example, during an immune response, it is expected that resting immune cells (relatively inert) become activated, and this requires a change in metabolism – metabolic reprogramming crucial for immune response to pathogens. Immune cells rely on the combination of different metabolic pathways – glycolysis, aerobic glycolysis, tricarboxylic acid (TCA) cycle, and oxidative phosphorylation (OXPHOS) – to achieve function regulation (65, 66). Pro-inflammatory immune cells present a glycolytic phenotype – they are engaged in aerobic glycolysis. This phenotype allows the production of carbon intermediates and biosynthetic precursors vital for amino acids, nucleotides, and lipids synthesis that sustains active proliferation and production of inflammatory mediators, and allows cells to adapt to adverse conditions such as hypoxia (62). But immune cells can also use the TCA cycle and OXPHOS, especially when quiescent or non-proliferative and their main requirements are energy and longevity (62). Immunometabolism of different immune cell subsets was reviewed in (67–70).

It is recognized that both chronological age and age-associated symptoms – systemic accumulation of SC – may impact immunometabolism. In fact, SC directly exacerbates various age-associated symptoms and contributes to immune cell dysfunction and immunometabolism imbalance. SC has distinctive features: permanent growth arrest; enlarged, vacuolated, and flattened shape; multinucleated with irregular nuclear forms; gradually more irresponsive to mitogens and growth factors; metabolically active; and increased senescence-associated β-galactosidase activity (SA-β-gal) (71, 72). Both CD4^+^ and CD8^+^ T-cells can acquire a senescent phenotype (_*SEN*_T-cell), marked by loss of CD27 and CD28 and acquisition of KLRG1 and CD57 expression. These CD27^–^ CD28^–^ KLRG1^+^ CD57^+^_*SEN*_T-cells show shortened telomeres, DNA damage responses, constitutive activity of MAPK, and exhibit SASP, which differentiate them from effector T cells (71, 73). So, _*SEN*_T-cells secrete cytotoxic mediators, express natural killer cell receptors (NKR), and recognize stress ligands induced by inflammation/infection. These features highlight the importance of _SEN_T-cells in immunopathology, especially if related to aging and inflammation/infection (e.g., COVID-19) (71, 72).

When SC accumulates in tissues, a metabolic switch occurs. From a metabolic point of view, SC is more glycolytic than their non-senescent counterparts; and this increased reliance on glycolysis supports SASP production (74). Extensive activation of glycolytic pathways results in alterations in metabolite concentrations, pH, and redox homeostasis – which further negatively impacts the immune response (36). mTOR, AMP-activated protein kinase (AMPK), and sirtuins have been studied as potential targets for aging (75–77). In fact, these three metabolic and nutrient sensing pathways, involved in the detection of nutrient scarcity and catabolism, are dysregulated during aging, connecting metabolic dysfunction to inflammaging (27, 36). So, targeting dysregulated metabolism or senescence seems to be interesting to improve aged immune responses, especially when facing infectious agents. Regarding _*SEN*_T-cells, targeting IL-7 as a growth factor for naïve T cells, the use of checkpoint inhibitors to improve T-cell response in aging, and inhibition of MAPK and their interaction with nutrient-signaling pathways, have been pointed has potential targets for immunosenescence (27).

Genetic diseases have a significant impact in addition to the cellular and molecular changes brought on by immunosenescence (8). In fact, we can assume that the various environmental elements that affect immune function as we age, such as nutrition and the microbiome, act by inducing a physiological response that eventually becomes visible as a result of altered gene expression (8, 78). All biological processes are known to be regulated by epigenetic mechanisms, which manage gene transcription and translation. Disruptions in these mechanisms can cause an abnormal rise or fall in protein synthesis in aging cells (79). DNA methylation, histone changes, and microRNAs are some of the epigenetic mechanisms that are present in aging immune cells from the innate and adaptive immune systems (79). One of the important epigenetic factors known to regulate gene expression is DNA methylation, which is primarily restricted to CpG-rich regions grouped in the promoter region of the majority of genes (80). Several immune system cells have been found to have changes in DNA methylation with aging, which affect the proliferative capacity and functionality of these cells (8). For instance, decreased DNA methylation affects the phenotypic plasticity of monocytes, the generation of TNF-α by monocytes and peripheral blood mononuclear cells (PBMCs), the cytotoxicity of NK cells, Treg formation and function, and B cell activity (81). Additionally, affecting innate and adaptive immune responses is histone modification. Aged immune cells have been shown to exhibit cellular defects that result in the generation of pro-inflammatory cytokines including IL-6 and TNF-α as well as chronic inflammation (82). In animal models, senescence of T-cells and altered gene expression have both been linked to decreased histone methylation and acetylation (8). Regarding micro-RNAs, altered production of IL-6, TNF-α, and CXCL9 in dendritic cells and macrophages has been connected to the expression of several miRNAs in the aged (8, 79). B and T cell functionality can also be impacted by micro-RNA; for instance, lower expression of miR-92a in aged people has been linked to a reduction in the number of naive CD8^+^ T-cells, which results in terminal differentiation and exhaustion (83).

### Immunosenescence and nutrition

**FIGURE 2:**
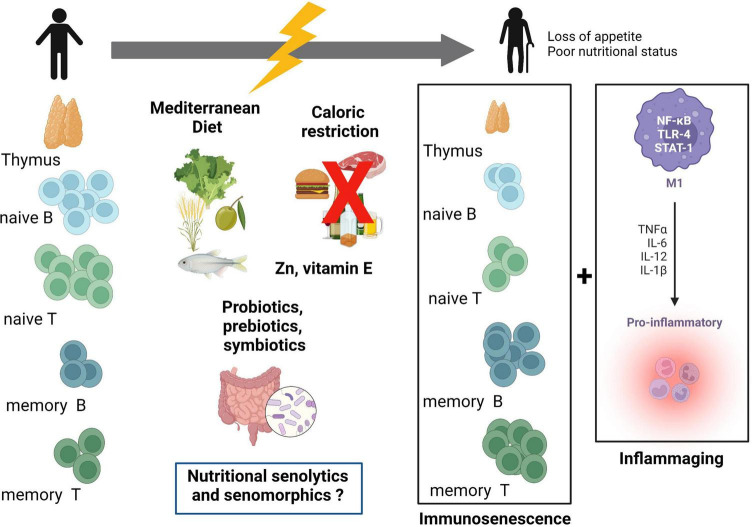
Relation between immunosenescence, inflammaging, and nutrition. Immune cells and the thymus are affected during the aging process. Thymic atrophy, reduction of naïve B- and T-cells, and increase in memory B- and T-cells are hallmarks of immunosenescence. These events together with increased low-grade inflammation (inflammaging) may constitute potential targets for nutritional interventions. Mediterranean diet, caloric restriction, vitamin E, zinc, and probiotics are possible modulators of immunosenescence and inflammaging. Nutritional senotherapeutics may be an additional form of targeting aging, as part of a personalized nutrition approach. TLR-4, toll-like receptor-4; STAT-1, signal transducer and activator of transcription 1; M1, macrophage M1 subpopulation. Created with BioRender.com.

Nutrition is an interesting way of modulating immune cell metabolism; indeed, nutrients play an important role in controlling immune responses. Some pathological conditions (like cancer and infection) highlight the concept of unequal nutrient availability to immune cells, where competitive nutrient uptake in immune microenvironments seems to be of major relevance for immune responses, especially in regulating immune cells’ fate (13, 84, 85). The study of the role of nutrients in immunometabolism has increased in recent years. If immunometabolism is dysregulated with immunosenescence, nutrition has a potential interest in reverting hallmarks of aging associated with metabolism and inflammation (85). Figure 2 presents the dynamic relationship between nutrition and immunosenescence, highlighting nutrition-related strategies to address aging-related alterations in immune cells. Within this topic, interesting studies have been carried out with micronutrients such as vitamin E and Zinc, with dietary patterns such as the Mediterranean Diet and even with caloric restriction (86). Studies on aged mice and people showed that supplementing with vitamin E improved immunological responses, especially in those with weakened immune systems (87, 88). Additionally, prior research found that Zn supplementation improved immunological function (89, 90). However, its favorable effects on the immunological response have not been consistently shown, notably in investigations involving humans. Therefore, additional research is required to ascertain the effectiveness of zinc in preventing infection in the elderly. Fresh fruits, which are rich in vitamins and nutrients, and a high intake of plant-based foods are characteristics of the traditional Mediterranean diet. MD has a very low saturated fat content and is enriched by polyunsaturated fatty acids, particularly omega-3 fatty acids. The potential protective effect of this diet on immune function was demonstrated by a wide range of *in vitro* and *in vivo* studies (91–94). The majority of research demonstrating the beneficial effects of CR on immune response was then carried out on rodents, namely mice. The impact of CR was also studied in non-human primates and for the most part, they have generated similar results to those seen in rodent studies regarding the effect on immune response and several other age-related disorders (95). Recent findings from studies on humans indicate that these effects might also be relevant to people (96). Vitamin E and Zinc exert their functions mainly by reducing oxidative stress. In addition to the beneficial effects already described for the Mediterranean Diet, this dietary pattern has been linked to the downregulation of cell adhesion molecules such as Intercellular Adhesion Molecule (ICAM)-1 and Vascular Cell Adhesion Molecule (VCAM)-1, and proinflammatory mediators such as CPR and IL-6 (97–99). Caloric restriction, associated to improved lifespan and healthspan, showed potential by downregulating IL-1β, IL-6, and TNF-α, and in reducing inflammation (100–102). Another class of potential modulators of SASP and immunometabolism are the so-called senotherapeutics, particularly those considered nutritional senotherapeutics (14). This will be detailed below. Nevertheless, strategies to address immune “rejuvenation” in older individuals are needed, especially if engaged as targeted/tailored interventions inducing a safe “rejuvenation” of immune status rather than a non-specific “rejuvenation,” which could be counterproductive (27).

### Introduction to senotherapeutics: Senomorphics and senolytics

As stated earlier, SC and SASP can be viewed as targets for specific strategies to improve lifespan and healthspan. These strategies can include the selective killing of SC or selective blockade of SASP, through natural or synthetic products – called senotherapeutics. Senotherapeutics are divided into senolytics and senomorphics, both aiming at the elimination or the delay of cellular senescence, the aging process, and consequently age-associated pathologies. Clinical evidence on senotherapeutics remains scarce and the short- and long-term side effects remain unidentified. An extensive review of senotherapeutics can be found in (21, 22).

Senomorphics are small molecules that promote SASP blockade without cell killing, and indirectly hampering the senescence phenomenon. Their targets are pathways related to SASP expressions, such as the p38MAPK, PI3k/Akt, mTOR, and JAK/STAT pathways, transcription factors, such as NF-κB, C/EBP β, and STAT3, and, neutralization of the activity and function of IL-1α, IL-8, and IL-6 (specific SASP factors) with specific antibodies. In parallel, senolytics are small molecules that can selectively eliminate SC through apoptosis; delaying, preventing, or even reversing the senescence process and increasing healthspan (and perhaps lifespan). Even small reductions in the number of SC have been associated with the reduction of SC’s load and to reversion of age-associated symptoms (such as frailty and sarcopenia). In Table 2, natural and synthetic senotherapeutics are summarized. Natural senomorphics and senolytics from food sources have been called “nutritional senomorphics” and “nutritional senolytics”; whose interest will be discussed below.

**TABLE 2:** Summary of natural and synthetic products used as senotherapeutics.

	Senomorphics	Senolytics
**Natural**	Rapamycin* *Resveratrol* *Kaempferol* *Apigenin* *EGCG*	*Quercetin* *Fisetin* *Piperlongumine* EF24 curcumin analog *Curcumin* Ouabain* Digoxin*
**Synthetic**	Metformin* Ruxolitinib* Cortisol & Corticosterone* Loperamide & niguldipine * KU-60019 NBD peptide Mmu-miR-2910-3p	Dasatinib * ABT-263 ABT-737 A1331852 UBX1325 UBX0101 P5091 Geldanamycin Tanespimycin Alvespimycin * FOXO4-DRI Panobinostat * MitoTam

Nutritional senomorphics and nutritional senolytics are italicized. *FDA approval for clinical use.

### Nutritional senotherapeutics: From theory to practice

The majority of anti-senescence properties attributed to nutritional senotherapeutics rise from cellular and animal models, but some evidence from clinical trials is emerging. Senescent human fibroblasts, aged rat kidneys, senescent human IVD cells, senescent Ercc1-/- murine embryonic fibroblasts, senescent human cells *in vitro*, progeroid and aged mice *in vivo*, aged and hypercholesterolemic mice models, and senescence-accelerated mouse models are the main models used to study the effect of these compounds on senescence (20).

Nutritional senomorphics promoting SASP blockade without cell killing are resveratrol, kaempferol, apigenin, and EGCG. Resveratrol is a flavonoid, abundant in fruits and red wine (21), characterized by its anti-inflammatory and antioxidant properties. Its senomorphic properties, observed in different cellular and animal models, consist of the inhibition of the NF-κB pathway (a major SASP regulator, as presented earlier) and the activation of the Nuclear factor-erythroid factor 2-related (Nrf2) transcription factor (a negative regulator of NF-κB) (103–105). Two other flavonoids with antioxidant activity found in plants, fruits, vegetables, and green tea have been identified as potent senomorphics: apigenin and kaempferol. These nutritional senomorphics inhibited NF-κB activity inhibition through the IRAK1/IκBα signaling pathway in cellular models (106–108). Also, apigenin reduced SASP levels to decrease NF-κB activity initiated by IL-1α through reduced IRAK1/IRAK4/p38MAPK phosphorylation in cellular models (107). Apart from flavonoids, green tea is rich in EGCG, a phytochemical with potent radical scavenger activity. Studies have shown that EGCG may suppress senescence through the inhibition of the stress-induced PI3k/Akt/mTOR signaling and the inhibition of AMPK activation. Also, it was demonstrated that this phytochemical can inhibit ROS, Cox2, NF-κB, and SASP factors (IL-6 and TNF-α) expression, as part of its antioxidant activity (109, 110).

Nutritional senolytics that selectively eliminate SC through apoptosis are quercetin, fisetin, piperlongumine, and curcumin (21). Quercetin is a plant flavonoid with antioxidant activity, whose senolytic activity has been revealed both *in vitro* and *in vivo*. Quercetin acts mostly via mTOR, NF-κB, PI3k/Akt, p53/p21/serpine, and HIF-1α pathways and has been related to extending organismal lifespan in some cellular and animal models (111, 112). In parallel, fisetin is also a bioactive flavonol found in vegetables and fruits, known for its antioxidant and anti-inflammatory activities. Senolytic properties of Fisetin are mainly due to the regulation of NF-κB and Nrf2 redox-sensitive transcription factors (113–115). Piperlongumine is a natural alkaloid amide with senolytic activity found in Piper species. In target cell types, piperlongumine showed an ability to inactivate OXR-1 and a ROS-reducing enzyme, driving apoptosis in a ROS-independent way (116–118). Curcumin is an extract from turmeric with frequently associated with anti-inflammatory, antioxidant, anti-microbial, and anti-cancer properties. Cellular models have enlightened the senolytic properties of curcumin. Curcumin appears to increase the lifespan of *D. melanogaster* and *C. elegans* (119). Also, curcumin exerts its senolytic activity through the activation of Nrf2, reduction of NF-κB activation, and reduction of proinflammatory cytokines expression. Interestingly, this seems to be accompanied by decreased levels of IL-1β and TNF-α and enhanced levels of IL-10 (120–123).

**FIGURE 3:**
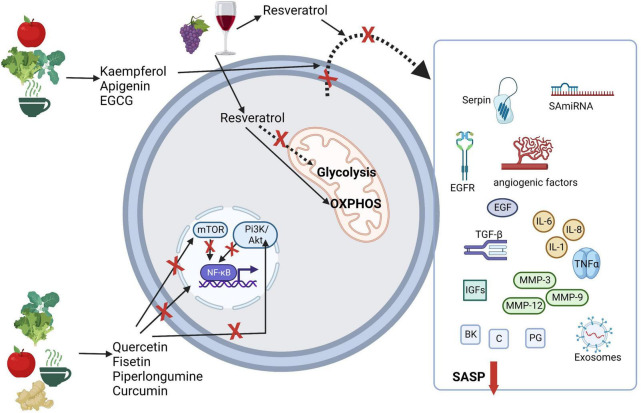
Possible mechanisms involving nutritional senolytics and senotherapeutics in aging. Akt, protein kinase B; BK, bradykinin; C, ceramides; EGCG, epigallocatechin-3-gallate; EGF, epidermal growth factor; EGFR, epidermal growth factor receptor; IGFs, insulin-like growth factors; IL, interleukin; MMP, matrix metalloproteases; mTOR, mammalian target of rapamycin; NF-kB, nuclear factor-κB; OXPHOS, oxidative phosphorylation; PI3K, phosphoinositide 3-kinases; PG, prostaglandin; SA-miRNA, senescence-associated microRNAs; TGF, transforming growth factor; TNF, tumor necrosis factor. Created with BioRender.com.

Considering immunometabolism, the suppression of the PI3k/AKT/mTOR pathway by flavonoids endorses the reduction of effector T-cell differentiation and induces regulatory T-cells (124). In this context of immunometabolism and immunosenescence, resveratrol is particularly interesting. As stated earlier, SCs namely senescent immune cells, like T-cells present a more glycolytic phenotype than their non-senescent counterparts (36, 125). Resveratrol seems to have the ability to increase mitochondrial activity and associated OXPHOS (126). Interesting results point to interruption of pro-inflammatory activity in CD4 + T-cells by resveratrol-mediated SIRT1 activation; and a low dose of resveratrol rapidly stimulated genotoxic stress in T-cells and concomitant activation of p53, leading to a metabolic reprogramming characterized by decreased glycolysis, increased glutamine consumption, and a shift to OXPHOS (126). These alterations resulted in enhanced effector function of CD4^+^ T-cells, and increased production of IFN-γ by naïve and memory CD4 + T-cells (126). Resveratrol’s potential as a health-promoting agent is so significant that it is used as a mimetic of caloric restriction both in cellular and animal studies (127). Caloric restriction, as presented in Figure 2, is also a promoter of the aging arrest. In Figure 3, we synthesized the possible mechanisms involving nutritional senotherapeutics in aging.

It seems clear that nutritional senotherapeutics appear to constitute a possibility to reverse the senescence process, either by SASP blockade and SC killing. Recent advances also demonstrate that some of these nutritional senolytics and senomorphics may modulate immune cell metabolism, counteracting the highly glycolytic phenotype and promoting OXPHOS (126). However, human studies devoted to elucidating these functions with a high degree of evidence are scarce. We searched MEDLINE, Scopus, and Embase, with no language restriction, but restricted to Clinical Trials, relating the specific senotherapeutic terms that are the subject of this review to the following MeSH Terms: (aging OR senescence OR immunosenescence) AND (immune system OR inflammation). This search is updated to 31 May 2022. In Table 3, we gathered the main concluded clinical trials performed in older individuals and assessed the effect of nutritional senotherapeutics on immunosenescence, particularly on cellular and humoral components and markers of inflammation and oxidative stress. The mainly nutritional senotherapeutics studied were resveratrol and curcumin. Although it should be noted that this is a very current topic, the data collected are very heterogeneous (mainly in terms of dosage) and the number of studies is very small. In the study by Harper et al. (128), resveratrol failed to improve inflammatory markers but decreased markers of oxidative stress. Regarding curcumin, the study from Kishimoto et al. (129) was the only one to show any effect, and it was confined to changes in white blood cells (WBC) composition and neutrophil-to-lymphocyte ratio (NLR).

**TABLE 3:** Clinical trials completed evaluating nutritional senotherapeutics on immunosenescence, cellular and humoral components, and markers of inflammation and oxidative stress in aged individuals.

	Study (Ref.)	Participants	Intervention			Outcomes	Results
			
			*Route*	*Daily dose*	*Timing*		
***Nutritional senomorphics***							
Resveratrol	Harper et al. (128)	Older adults with physical function limitations *N* = 60 (45 females) 71.8 ± 6.3 years old	Oral	500 mg resveratrol, 2 capsules daily + exercise; or 1000 mg resveratrol, 2 capsules daily + exercise *versus* placebo	12 weeks	*Inflammatory markers* VCAM-1; E-Selectin; IL-6 *Oxidative stress biomarkers* MPO; OxLDL; Plasma NO2^–^; Plasma NO3	*Inflammatory markers* - no differences between placebo and intervention *Oxidative stress biomarkers* - ↓ in Plasma NO2^–^ with higher dose
***Nutritional senolytics***							
Curcumin	NCT03085680, results published on February 2nd 2022 (https://clinicaltrials.gov/ct2/show/results/NCT03085680) Kishimoto et al. (129) Santos-Parker et al. (139)	Older adults at high risk of functional decline Older adults *N* = 40 (11 females) 65–75 years old Middle-aged and older adults *N* = 39 (18 females) 45–74 years old	Oral Oral Oral	1,000 mg curcumin, 2 capsules daily *versus* placebo 90 mg curcumin (curcuRouge*TM*), 2 capsules daily 400 mg curcumin, 1 capsule daily *versus* placebo	12 weeks 4 weeks 12 weeks	*Inflammatory markers* IL-6 WBC composition and NLR *Inflammatory markers* IL-6; TNF-α; PCR *Oxidative stress biomarkers* TAS; OxLDL; glutathione peroxidase	Inflammatory markers - no differences between placebo and intervention ↓ WBC and neutrophils count ↓ NLR ↑ Lymphocyte ratio No differences

IL, interleukin; MPO, myeloperoxidase; NLR, neutrophil-to-lymphocyte ratio; NO2^–^, plasma nitrite; NO3^–^, plasma nitrate; OxLDL, oxidized low-density lipoprotein; TAS, total antioxidant status; VCAM, vascular cell adhesion molecule; WBC, white blood cell. Uparrow represent the increase. Downarrow represent the descrease.

However, research in this field continues. At least three studies are registered at ClinicalTrials.gov, aiming to enlighten knowledge on nutritional senotherapeutics and immunosenescence: NCT04994561 is not yet recruiting and consists of a phase 1 study on the effect of a combination of resveratrol, quercetin, and fisetin; NCT03675724 (AFFIRM-LITE) is recruiting at the moment and is a phase 2 study on the effect of quercetin. NCT05234203 is a prospective, interventional, single-arm, open-label pilot study, that intends to study the effect of HTB Rejuvenate^®^, a supplement of quercetin, rutin, luteolin, and hesperidin. All promise to evaluate the effects on immunosenescence, cellular and humoral components, and markers of inflammation and oxidative stress Table 4. There are still many unanswered questions about the role that nutritional therapeutics can play in both preventing and reversing immunosenescence, particularly their interest as modulators of the metabolism of immune cells (specifically _*SEN*_T-cells). While it is interesting that clinical trials already exist to test the effects of some nutritional senotherapeutics on immune cell metabolism, more studies are needed.

**TABLE 4:** Ongoing clinical trials aiming to evaluate nutritional senotherapeutics on immunosenescence, cellular and humoral components, and markers of inflammation and oxidative stress in aged individuals.

Trial number	Nutritional senotherapeutics	Type of study	Intervention	Outcomes	Situation
NCT04994561	Resveratrol + quercetin + fisetin	Phase 1	1,500 mg glucosamine + 600 mg nicotinamide riboside and + 500 mg *trans*-resveratrol, for 50 weeks	Senescent cell-cycle arrest (MMP-9 measurement)	Not yet recruiting
NCT03675724, AFFIRM-LITE	Quercetin	Phase 2	Fisetin 20 mg/kg/day, orally for 2 consecutive days	Inflammatory markers	Recruiting
NCT05234203	Quercetin + rutin + luteolin + hesperidin	prospective, interventional, single-arm, open-label pilot study	2 capsules of HTB Rejuvenate^®^ (600 mg of quercetin, rutin, luteolin and hesperidin per serving), twice a day for 90 days	Numbers of T-cell subsets and granulocytes	Not yet recruiting

MMP, matrix metallopeptidase.

### Clinical perspective and future directions – precision nutrition approach

**FIGURE 4:**
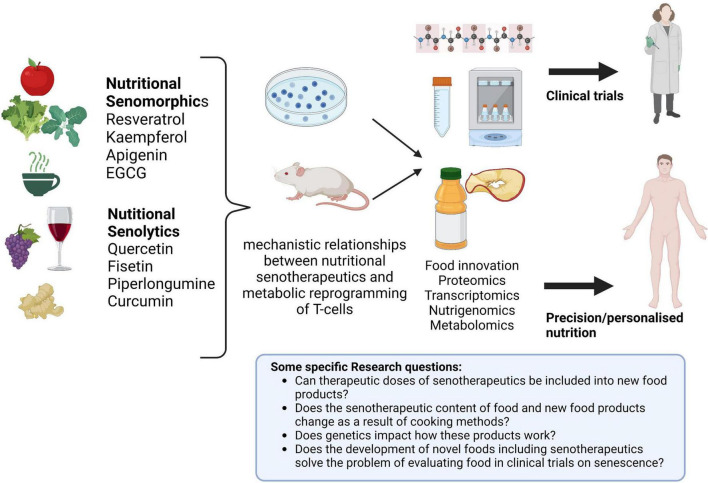
Future approaches in nutritional senotherapeutics. Cellular and animal models may contribute to elucidating the role of nutritional senotherapeutics in T-cell reprogramming and serve as a basis for the development of new food products that can be tested in clinical trials and subsequently integrate personalized nutrition schemes. Created with BioRender.com.

Clinical nutrition practice faces new challenges as the fields of personalized nutrition and immunometabolism expand and call for further research. Personalized host-directed therapies (HDT) are increasingly supported by scientific evidence, calling for a patient-specific manner of delivering nutrients and nutritional/food counseling (130, 131). Aligned with this is the emerging era of “big data” and developments in almost every field (from health to economics and technology) that dominates the modern age. We are now in a post-genomic era marked by new terms and definitions: “personalized nutrition,” “nutrigenomics,” “metabolomics,” and “foodomics” (132–134). These novel domains should be accounted for when public health, wellbeing, health span, lifespan, and knowledge are addressed. Regarding nutritional senotherapeutics and their possible use as modulators of immunosenescence and immunometabolism, there is still work to be done. Although data from cellular and animal studies are difficult to extrapolate to humans, they may elucidate mechanistic relationships between nutritional senotherapeutics and metabolic reprogramming of T-cells. Human studies concluded or on-going preferably test nutritional senotherapeutics in capsules for oral administration (which is understandable given the difficulties associated with concealment when food is used), but this brings an additional problem to this discussion, as it will always be preferable to consume a food versus supplements/drugs. However, consuming these products simply through food makes it more challenging to obtain therapeutic effect dosages. It should be remembered that some of these natural products are spices, like curcumin, used in small doses and intermittently, or components that are only very minutely present in the original foods (e.g., quercetin and kaempferol content in capers) (135). Additionally, it is important to keep in mind that alcoholic beverages may not be recommended in specific clinical circumstances when thinking about resveratrol sources. From other perspectives, there is also the problem of supplement composition and safety, which is not well controlled in many nations (136). All of these factors highlight the necessity for a fresh approach to the research on nutritional senotherapeutics, probably following different directions. In the field of food innovation (137), integrating these nutritional senotherapeutics into novel food items may be a way to improve research and increase food options sustained in the scientific background that can be easily integrated into individual eating patterns (Figure 4).

## Conclusion

Modulation of immune system metabolism and senescence by nutritional senomorphics and senolytics may be beneficial as future strategies to address aging. Based on cellular and animal studies, nutritional senotherapeutics appear promising in this field. Ongoing clinical trials should evaluate metabolic reprogramming of T-cells along with changes in cellular and humoral components, and markers of inflammation and oxidative stress. Well-designed clinical trials evaluating the effect of nutritional senotherapeutics on these outcomes are urgently needed. Nutrition science can improve this field, making use of gastrotechnics and molecular gastronomy knowledge to develop new food products enriched in these nutritional senotherapeutics, that can be tested in clinical trials and subsequently included in a personalized nutrition scheme (137, 138).

## Author contributions

RR and RS conceptualized the manuscript. RR reviewed all available data and clinical trials, drafted, revised the manuscript, and draw the images. CL reviewed all available data and clinical trials and drafted and revised the manuscript. AM drafted and revised the manuscript. PP, JM, and RS reviewed all available data and clinical trials and revised the manuscript. All authors finalized and approved the submitted version..
